# Developing a “Caregiving Record” for Home Health Patients with Dementia

**DOI:** 10.1002/alz70858_097639

**Published:** 2025-12-24

**Authors:** Julia G Burgdorf, Margaret V. McDonald, Kathryn Bowles

**Affiliations:** ^1^ Center for Home Care Policy & Research, New York, NY, USA; ^2^ VNS Health Center for Home Care Policy and Research, New York, NY, USA

## Abstract

**Background:**

Home health care (HH) provides skilled nursing, therapy, and aide services in the home. One‐third of HH patients have diagnosed dementia and these individuals rely heavily on support from family and unpaid caregivers during HH care. Yet, caregiving context is not systematically assessed in HH, presenting a barrier to high‐quality care for patients with dementia. Partnering with a major HH agency, we develop a “caregiving record”: an assessment tool to record relevant caregiving context information and workflow to share this data with frontline clinicians.

**Method:**

Under the guiding principles of participatory ergonomics, a user‐centered approach to intervention development, we employed a mix of formative and qualitative research methods to identify and refine a set of assessment items, test assessment content validity, and determine the preferred process for capturing and sharing this information within existing clinical workflows. Research activities included informational interviews with HH agency administrators, structured focus groups with HH clinicians (*n* = 18) and caregivers for patients with dementia (*n* = 10), recurring co‐design workgroup with administrators and clinicians (*n* = 13), and cognitive interviews with clinicians (*n* = 10) and caregivers (*n* = 11).

**Result:**

The final “caregiving record” assessment includes 10 items across three domains: (1) Primary caregiver identity and availability, (2) Caregiving network composition and tasks, and (3) Primary caregiver role‐related strain. The assessment received a Scale‐Content Validity Index score of 0.89 from clinicians and caregivers, indicating strong content validity. Using existing clinical workflows and IT infrastructure, the co‐design workgroup identified the preferred workflow as: fielding assessment items via phone call to caregivers 2 days after HH start of care and capturing results in a HIPAA‐compliant survey platform, then leveraging existing data management programs to automatically upload this information as a formatted care coordination note in the patient record.

**Conclusion:**

Providing a “caregiving record” for HH patients with dementia is a promising opportunity to improve HH care delivery for this population, with both clinicians and caregivers enthusiastic about such a process. Initial evaluation of the caregiving record demonstrates strong content validity and feasibility within a “real‐world” clinical setting.

